# Evaluation of DNA extraction methods from clinical *Mycobacterium tuberculosis* primary liquid culture for whole-genome sequencing

**DOI:** 10.1186/s12864-026-12812-w

**Published:** 2026-05-19

**Authors:** Emilyn Costa Conceição, Mishka Haffejee, Felicia Wells, Brendon Mann, Tim Heupink, Astrid Paulse, Jennifer Williams, Anzaan Dippenaar, Yonas Ghebrekristos, Megan Burger, Vincent Rennie, Miguel de Diego Fuertes, Ilham Al-Talib, Nabila Ismail, Melanie Grobbelaar, Elizabeth Maria Streicher, Túlio de Oliveira, Gian Van der Spuy, Annelies Van Rie, Robin Warren

**Affiliations:** 1 South African Medical Research Council Centre for Tuberculosis Research, Division of Molecular Biology and Human Genetics, Faculty of Medicine and Health Sciences, Stellenbosch University, Cape Town, South Africa; 2 Tuberculosis Omics Research Consortium, Department of Family Medicine and Population Health, Global Health Institute, Faculty of Medicine and Health Sciences, University of Antwerp, Antwerp, Belgium; 3 National Health Laboratory Service Green Point, Cape Town, South Africa; 4 Centre for Epidemic Response and Innovation (CERI), School of Data Science and Computational Thinking, Stellenbosch University, Cape Town, South Africa

**Keywords:** Whole-genome sequencing, DNA extraction, Diagnostics, Commercial kits, Clinical primary MGIT culture

## Abstract

Whole-genome sequencing (WGS) has potential for determining the complete drug-resistance profile of clinical *Mycobacterium tuberculosis* (*Mtb*) strains. Cetyltrimethylammonium-bromide (CTAB), the conventional method for *Mtb* DNA extraction, is labour-intensive and difficult to implement in routine laboratory settings. This study evaluated the performance of commercial kits to extract DNA from clinical primary liquid cultures (CPC). *Mtb-*positive decontaminated sputum sediments were pooled and used to inoculate mycobacteria-growth-indicator tubes (MGIT). Positive non-contaminated MGIT cultures were pooled and aliquoted to generate 10 technical replicate isolates for DNA extraction by CTAB method with (+) or without (-) RNAse and nine DNA extraction commercial kits (+/-modifications): Zymo-DNA Clean and Concentrator, Zymo-Quick-DNA Fungal/Bacterial (+ lysozyme-digestion), InstaGene (IGM) +/-RiboLyser Homogeniser (RH), GenoLyse (+/-precipitation), FluoroLyse (+/-precipitation), PrepGEM-Bacterial (+/-precipitation), NucleoSpin-Tissue, NucleoMag-Pathogen, and Gene-Xpert buffer (+ precipitation or +purification). Genomic libraries for WGS were generated using the Illumina DNA-Prep Kit and evaluated for quality using NanoDrop, Qubit DNA ds/HS and TapeStation assays. Ten performance parameters were evaluated out of a score of 5: quantity of total dsDNA, DNA purity ratio A260/280 and A260/230, PCR amplifiability (targeting the *pncA* gene, 615 bp), concentration of genomic libraries (ng/µl), library fragment size (bp), mapped percentage, median coverage, turnaround time and cost. IGM/RH showed the highest overall performance, followed by IGM, CTAB/RNase, GenoLyse+precipitation, and FluoroLyse+precipitation. Commercial methods achieved sequencing results comparable to CTAB while requiring shorter processing times and lower costs. Simplified commercial extraction methods can produce DNA suitable for WGS from CPC samples while reducing processing time and cost compared with CTAB, supporting their use in routine TB workflows.

**Supplementary Information:**

The online version contains supplementary material available at 10.1186/s12864-026-12812-w.

## Introduction

Comprehensive drug susceptibility testing for surveillance and patient management results is a slow and expensive process in most settings. Whole-genome sequencing (WGS) of *Mycobacterium tuberculosis* (*Mtb*) is a promising method for the diagnosis of the complete drug resistance profile of an *Mtb* isolate through a single assay [1] and thereby enhances precision medicine for patient care [2].

In an ideal scenario, the optimal approach would involve performing WGS on DNA extracted directly from a clinical specimen, such as sputum, but currently this is technically challenging and requires the use of expensive *Mtb* DNA enrichment methods [3]. Therefore, WGS of *Mtb* requires an initial culture step, often followed by a sub-culture, to increase the *Mtb* biomass thereby enriching for *Mtb* DNA present in the clinical sample. Culture also ensures that sufficient *Mtb* DNA of high quality, quantity, and integrity is isolated after extraction. Unfortunately, this process takes weeks to months, limiting its use for real-time patient management [4].

In 2013, the first report on the use of WGS of DNA extracted directly from a mycobacteria growth indicator tube (MGIT) to investigate a patient with extensively drug-resistant TB was published [5]. In the past decade, several studies have used DNA extracted from MGIT cultures to perform WGS [6–18]. In 2015, Votintseva et al. developed a simple and inexpensive method for extracting purified mycobacterial DNA for WGS from a MGIT culture in approximately three days (2 to 5 days; interquartile range) after the MGIT tube flagged positive [18], later proposed to be called clinical primary liquid (MGIT) cultures (CPC) [19].

Briefly, the procedure includes decontaminating clinical samples before inoculation into MGIT to minimize contamination from naso/oropharyngeal microbiota. Subsequently, a saline wash is employed to reduce human DNA contamination in the positive *Mtb* MGIT cultures before DNA extraction. For DNA extraction, *Mtb* possesses a unique, lipid-rich cell wall that makes it inherently resistant to lysis, posing a significant challenge for DNA extraction. Consequently, conventional kit-based DNA extraction methods, such as the QIAamp DNA Mini kit (Qiagen, Hilden, Germany) and QuickGene DNA tissue kit S for QuickGene-Mini80 (Kurabo, Osaka, Japan), which often lack an intensified lysis step specifically designed for Mtb, prove less effective than the proposed methods.

Unfortunately, the ethanol precipitation method, while recommended, is labour-intensive and challenging to implement in routine laboratory settings, particularly in areas with a high burden of TB [18]. The same applies to the Cetyltrimethylammonium bromide (CTAB) method [20], the most commonly used method for *Mtb* DNA extraction. These methods, while effective, are not easily amenable to automation, which is crucial for achieving high throughput and efficiency in routine diagnostics, especially in high TB burden settings. Therefore, there is a critical need for the development and implementation of simplified automated DNA extraction methods that can streamline workflows and improve turnaround times in resource-constrained laboratories labour-intensive and challenging to implement.

To improve the practicality of WGS for diagnostic purposes in TB laboratories situated in high-burden TB countries, the performance of various commercial kits as alternatives to the labour-intensive CTAB method were assessed by comparing these alternatives in terms of DNA yield, purity, suitability for downstream applications, turnaround time, and cost-effectiveness when extracting DNA from CPC.

## Materials and methods

### Preparation of aliquots of pooled MGIT cultures and DNA Extraction pre-treatment

To minimize biological variability and enable controlled comparison of extraction methods, decontaminated sputum sediments obtained from multiple individuals with TB were pooled prior to culture. This approach ensured that all evaluated DNA extraction methods were applied to a standardized input material, allowing differences in downstream performance to be attributed primarily to the extraction protocol rather than inter-patient variability.

**Fig. 1:**
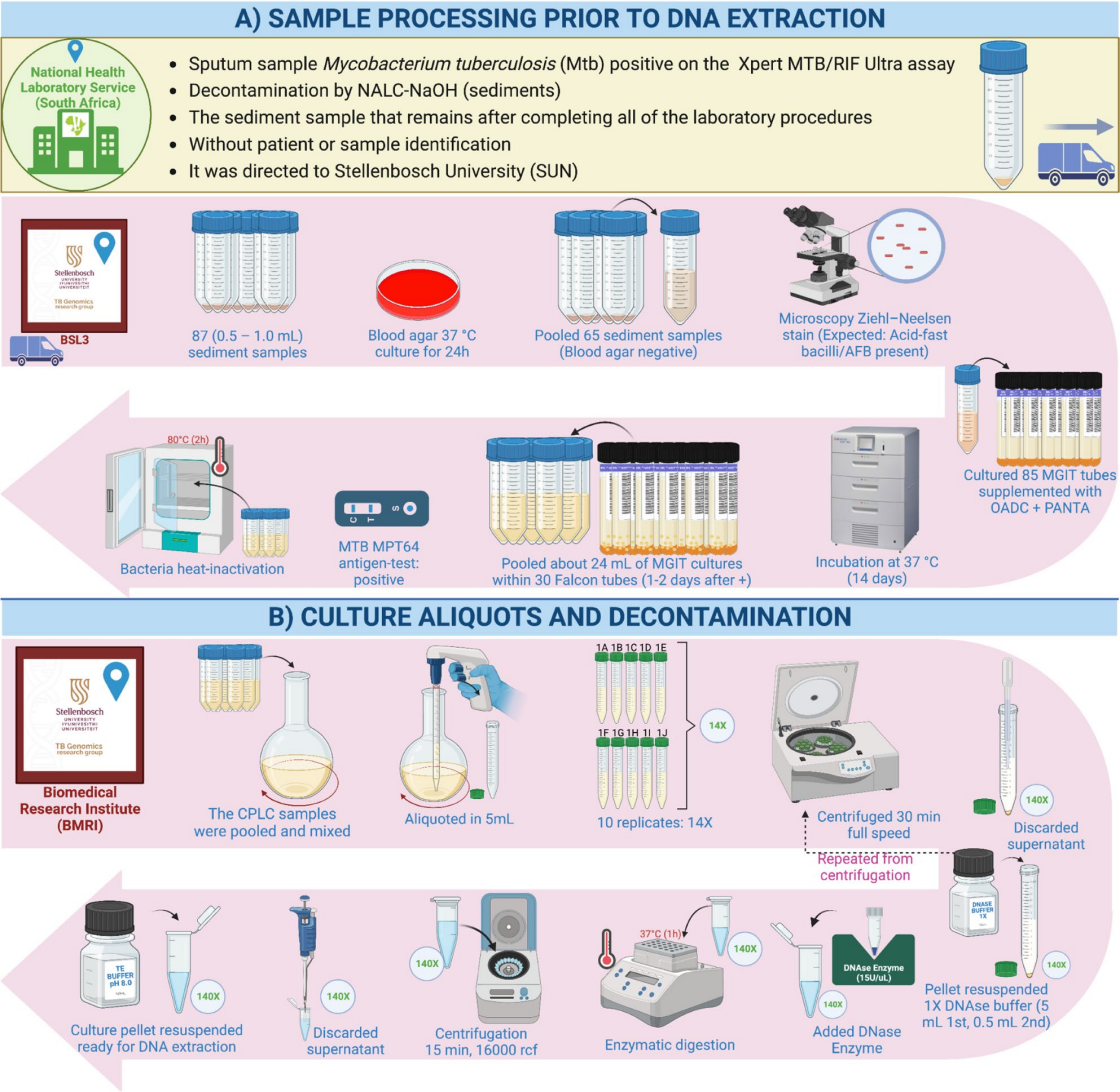
Sample processing workflow for generating biological technical replicates of Mycobacterium tuberculosis clinical primary liquid-MGIT cultures (CPC). The workflow includes: (**A**) Biosafety Level 3 (BSL-3) procedures involving decontaminated sediment pooling, culture, quality control, and bacterial inactivation, followed by (**B**) Biosafety Level 2 (BSL-2) procedures for culture aliquoting and pre-washing prior to DNA extraction

The sample set consisted of 65 NALC-NaOH decontaminated sputum sediments (0.5–1.0 ml) that were *Mtb* positive on the Xpert MTB/RIF Ultra (Cepheid, Sunnyvale, USA) by routine diagnosis at the National Health Laboratory Service in Green Point, Cape Town. The remaining anonymised sediment was shipped to the Biosafety Level 3 (BSL3) laboratory at the Biomedical Research Institute at Stellenbosch University. Sediments were cultured on blood agar at 37 °C for 48 h to exclude samples with high levels of contaminating bacteria. Blood agar–negative sediments were then pooled and examined microscopically using the Ziehl–Neelsen method. From the pooled sediments, 0.5 ml aliquots were used to inoculate 85 MGIT tubes supplemented with BD MGIT™ PANTA™ Antibiotic Mixture (Becton Dickinson, Franklin Lakes, USA). When the MGIT cultures flagged positive (Growth Index = 75), the cultures remained in the MGIT instrument at 37 °C for 24 h for extra incubation, followed by heat-inactivation at 80 °C for 2 h and removed from the BSL-3 to a general Biosafety Level-2 (BSL-2) laboratory (Fig. 1A).

### Pre-treatment Before DNA Extraction

The heat-inactivated culture was pooled (705.5 ml) followed by manual homogenization before 5 ml aliquots were transferred to 140 Falcon tubes and centrifuged at 3220 rcf for 30 min at room temperature using an Eppendorf 5810R centrifuge. The supernatant was carefully discarded using a 2 ml Pasteur pipette (Sigma-Aldrich, St. Louis, USA). The pellet was resuspended by adding 5 ml of 1x DNase buffer (prepared in-house from a 10X stock as per published protocol https://zenodo.org/records/18981453 [20], followed by homogenization using a vortex for 10 s to disperse the cells. The 15 ml tubes were centrifuged again at 4,000 rcf for 30 min at room temperature, and the supernatant was discarded again carefully using the 2 ml Pasteur pipette, at this time, the remaining supernatant was removed with a P1000 pipette to avoid pellet loss.

The pellet was resuspended in 500 µL of 1x DNase buffer within the 15 ml tube and transferred to a 1.5 ml NoStick^®^ Hydrophobic Microtubes (Scientific Specialties Inc., Lodi, USA) (low-binding tube). For enzymatic digestion, 10 µl (15 U/µL, totalling 150 U/sample) of DNase I enzyme (Thermofisher, Waltham, USA) was added followed by incubation at 37 °C for 30 min whilst shaking to deplete extracellular DNA from the host and/or commensal organisms.

The samples were then incubated at 65 °C for 10 min to deactivate the DNase I enzyme. This was followed by centrifugation at 21,000 rcf Eppendorf 5418 (Eppendorf, Hamburg, Germany) for 20 min to pellet all cells again. The supernatant was removed using a P200 pipette. The pellet was resuspended in 200 µL of Tris-EDTA (TE) pH 8.0 buffer solution (Sigma-Aldrich, St. Louis, USA) until DNA extraction (Fig. 1B).

### DNA extraction methods

Fourteen different DNA extraction methods were compared, including nine commercial kits with or without modifications, and the *in-house* CTAB method. Each of these 14 approaches was performed in replicates of 10 times (*n* = 140) and the samples for each of these were labelled accordingly (1 A-1 J up to 14 A-14 J) (Fig. 1B). The 14 approaches were; (1) CTAB method for reduced volume samples; (2) CTAB method for reduced volume samples with RNAse; (3) Zymo Clean and Concentrate™-25 kit (Zymo Research Corporation, Irvine, USA); (4) Zymo Quick-DNA Fungal/Bacterial Miniprep (Zymo Research Corporation, Irvine, USA); (5) InstaGene Matrix (IGM) (Bio-rad laboratories, Hercules, USA) with modifications regarding volumes and time of incubation (6) IGM and mechanical lysis through a high-speed homogenizer (HSH) as proposed by Shea et al. (2017, 2021); (7) FluoroLyse (Hain Lifescience GmbH, Nehren, Germany) kit (8) GenoLyse (Hain Lifescience GmbH, Nehren, Germany) kit (9) the Xpert buffer followed by CTAB purification; (10) the Xpert MTB/RIF buffer (Cepheid, Sunnyvale, USA) followed by Ethanol precipitation 11), PrepGEM Bacteria kit (MicroGem, Southampton, UK) followed by Ethanol precipitation; 12) PrepGEM Bacteria kit per manufacturer’s instruction (12 A-12 J); 13) MN NucleoSpin Tissue kit (Macherey-Nagel, Düren, Germany) and 14) MN NucleoMag^®^ Pathogen kit (Macherey-Nagel, Düren, Germany).

Due to the large number of samples processed (14 extraction methods × 10 replicates), DNA extraction experiments were conducted across multiple working days. To minimize experimental variability, the same operators, equipment, and reagent batches were used whenever possible, and extraction methods were distributed across processing days to reduce potential batch effects.

#### CTAB DNA extraction method

To ensure comparability with workflows currently used in TB genomics laboratories, the CTAB protocol evaluated in this study reflects the operational version commonly implemented for Mtb DNA extraction from culture-derived samples [21]. Although more intensive CTAB-based protocols have been described [22], including the use of acidic CTAB buffers and prolonged hot chloroform incubation to enhance DNA purification from complex biological matrices, such approaches substantially increase processing time, workflow complexity, and biosafety risks. These constraints limit their practicality for routine diagnostic and genomic surveillance workflows in high-burden TB settings. Therefore, the CTAB protocol used here represents a pragmatic implementation-oriented benchmark against which simplified commercial extraction methods were evaluated.

The CTAB DNA extraction method [21] was performed with modifications from the original protocol for a reduced input volume, optimised for CPC and low biomass samples. The original protocol is considered the conventional method for *Mtb* DNA extraction. For the CTAB method, to each of 10 resuspended pellets 50 µl of 10 mg/ml lysozyme solution (Thermofisher, Waltham, USA) was added followed by overnight incubation at 37 °C using a shaking incubator, the ProBlot 12 Hybridization Oven (Labnet International Inc., Edison, USA). Thereafter, 35 µl of freshly prepared Sodium Dodecyl Sulphate (SDS) 10% (Sigma-Aldrich, St. Louis, USA) and 5 µl of proteinase K solution (10 mg/ml) ((Thermofisher, Waltham, USA) was added. At this point, 5 µl of RNAse (10 mg/ml) (Thermofisher, Waltham, USA) was added only for samples 2 A to 2 J.

The mixture was vortexed for 3 s and incubated at 65 °C with intermittent mixing for 10 min for enzymatic digestion. For each sample 50 µl of 5 M NaCl (Sigma-Aldrich, St. Louis, USA) was added followed by manual homogenization and the addition of 50 µl pre-warmed CTAB/NaCl solution (Sigma-Aldrich, St. Louis, USA) at 65 °C using a thermoblock Techne DB-2 A Dri-Block Heater (Akribis Scientific Limited, Hereford, UK). The samples were vortexed for 3 s until the solution became milky. The samples underwent incubation at 65 °C for 10 min. Following this, 375 µl of room temperature chloroform/isoamyl alcohol (Sigma-Aldrich, St. Louis, USA) was added and vortexed for 3 s, followed by centrifugation at 11,000 rcf for 8 min. At this step, the top (aqueous) phase was carefully aspirated into a new 1.5-2.0 ml NoStick^®^ Hydrophobic Microtubes (Scientific Specialties Inc., Lodi, USA) (low-binding tube). Thereafter, the chloroform/isoamyl alcohol step was repeated, and the aqueous phase was transferred to a new low-binding tube, followed by the addition of 1 µl glycogen (Thermofisher, Waltham, USA) for every 100 µL of liquid transferred.

Following, an equal volume of ice-cold isopropanol (Sigma-Aldrich, St. Louis, USA) was added and the sample was inverted manually before incubation at -20 °C for at least 1 h. Afterwards, the sample was centrifuged at 12,000 rcf for 30 min, the supernatant was removed with a P1000 pipette and left with about 50 µl of liquid to avoid resuspending the pellet. Thereafter, 1 ml of freshly prepared and ice-cold 80% molecular grade ethanol (Sigma-Aldrich, St. Louis, USA) was added, mixed manually and centrifuged again at 12,000 rcf for 30 min. The Ethanol wash step was repeated, and then completely removed and allowed to fully dry at room temperature overnight. Finally, the DNA pellet was resuspended in 25 µl of room temperature nuclease-free water (NFW).

#### Zymo DNA extraction kits

For the Zymo kit DNA extraction methods (Zymo Research, Irvine, USA) both the Zymo Clean and Concentrate™-25 kit (sample set from 3 A to 3 J) designed for recovery of ultra-pure DNA from enzymatic reactions, and the Zymo Quick-DNA Fungal/Bacterial Miniprep kit (sample set from 4 A to 4 J) designed for extraction of DNA from tough-to-lyse fungi, Gram (+/-) bacteria, algae, and protozoa was used. For both approaches, before starting the DNA extraction, an enzymatic digestion was performed using 50 µL of 100 mg/ml of lysozyme (Thermofisher, Waltham, USA), which was added to each tube and incubated overnight at 37 °C in a shaker incubator, the ProBlot 12 Hybridization Oven (Labnet International, Inc., Edison, USA). Thereafter, 50 µL of 2.5 mg/ml proteinase K (Merck, Germany) and 100 µL of 20% sodium dodecyl sulphate (Thermofisher Scientific, Waltham, USA) was added and incubated at 65 °C for 30 min using a thermoblock Techne DB-2 A Dri-Block Heater (Akribis Scientific Limited, Hereford, UK). The resultant lysate was then purified using the kit according to the manufacturer’s instructions kits [23].

#### InstaGene matrix

For the InstaGene Matrix (IGM) (Bio-rad laboratories, Hercules, USA) two approaches were used: (1) According to the manufacturer’s instruction with modifications regarding volumes and time of incubation for the set of samples from 5 A to 5 J, and (2) Using a combination of IGM and mechanical lysis through a HSH as proposed by [12, 13] Shea et al. (2017, 2021) (6 A to 6 J). For this experiment, the high-speed homogenizer Hybaid RiboLyser (HR) was used instead of the FastPrep. For all samples (5 A-5 J, 6 A-6 J), the tubes were centrifuged at 21,000 rcf for 20 min to pellet cells. After removing the supernatant, 150 µl of the IGM was added and incubated at 56 °C for 10 min. Four sterile 2 mm glass beads (Sigma-Aldrich, St. Louis, USA) were added to each tube, vortexed for 10 s and incubated at 100 °C for 10 min using a thermoblock Techne DB-2 A Dri-Block Heater (Akribis Scientific Limited, Hereford, UK). At this point, only for the set of samples 6 A-6 J, the HR was used for 20 s at a speed of 4.0 m/s for 4 cycles. Following, both sets of samples 5 A-5 J, and 6 A-6 J were centrifuged for 5 min at 12,000 rcf to separate the matrix from the DNA. The tubes were stored at 4 °C within the IGM matrix. Before any further processing, once removed from storage, samples were again centrifuged for 5 min at 12,000 so the supernatant containing the genomic DNA could be used.

#### Hain lifescience GmbH DNA extraction kits

The FluoroLyse and GenoLyse buffers are part of the Hain LiveScience (Nehren, Germany) kits for *Mtb* identification and drug resistance prediction based on the detection of mutations associated with drug resistance. After centrifuging the tubes at 21,000 rcf utilizing an Eppendorf centrifuge for 20 min to pellet cells, DNA extraction was performed using the FluoroLyse kit and the GenoLyse kit in a set of samples, 7 A-7 J and 8 A-8 J, respectively according to the manufacturer’s instructions except that the 95 °C incubation time of 5 min was increased to 10 min due to the smaller pellet size of the samples. The supernatant and pellet were not separated to prevent the loss of large amounts of DNA when discarding the pellet. After performing the DNA quality control (QC), an ethanol precipitation step was applied to de-salt and concentrate the solution to obtain higher DNA yields.

The ethanol precipitation was done as follows. The tubes were centrifuged at 21,000 rcf utilizing a centrifuge Eppendorf for 10 min, the supernatant (190µL) was transferred to a new 1.5 ml tube, and 19 µl of 3 M sodium acetate (Thermofisher, Waltham, USA) was added with 1 ml of a pre-cold at -20 °C 96% -100% Ethanol molecular grade. After vortexing for 3 s, samples were incubated at -20 °C for 1 h, centrifuged at 21,000 rcf at 4 °C, utilizing a for 10 min, followed by careful removal of the supernatant. To wash the pellet, 500 µL of 70% molecular grade ethanol was used without disturbing the pellet, and after incubation at room temperature for 1 min, centrifugation was performed at 21,000 rcf and 4 °C for 5 min. This was followed by an additional brief spin-down to collect the remaining liquid which was then removed. The samples were left to dry at room temperature until all remaining ethanol was evaporated and thereafter, the samples were in 20 µL of NFW.

#### Xpert buffer

The Xpert MTB/RIF assay buffer (Cepheid, Sunnyvale, USA) was substituted as the main DNA extraction buffer. After centrifugation at 21,000 rcf for 20 min to pellet cells, the supernatant was removed and the cells were resuspended in 600 µl of Xpert buffer, vortexed for 1 min, and incubated at room temperature for 15 min. This was followed by an additional incubation at 95 °C for 10 min using a thermoblock Techne DB-2 A Dri-Block Heater (Akribis Scientific Limited, Hereford, UK). Two approaches were tested in association with Xpert buffer generated lysate: CTAB purification (9 A-9 J) and ethanol precipitation (11 A-11 J) as previously described.

#### PrepGEM bacteria kit

For the PrepGEM Bacteria kit (MicroGem, Southampton, UK) two approaches were applied: (1) performed according to the manufacturer’s instructions for “DNA extraction from capsulated bacteria/colonies/biofilms” protocol followed by previously described ethanol precipitation (10 A-10 J), and (2) performed according to the manufacturer’s instructions with additional purification (12 A-12 J). The tubes were centrifuged at 21,000 rcf utilizing a centrifuge Eppendorf for 20 min to pellet cells and resuspended the pellet in the extraction mixture provided by the kit (1 µL PrepGEM, 1 µL lysozyme, 10 µL 10X Green+ buffer and 88 µL NFW). The samples underwent the following incubation order: 37 °C for 15 min, 75 °C for 10 min and 95 °C for 5 min using a thermoblock Techne DB-2 A Dri-Block Heater (Akribis Scientific Limited, Hereford, UK). The samples were vortexed for 10 s, followed by a spin down for 1 min before using the extracted DNA.

#### Macherey-Nagel DNA extraction kits

For the MN NucleoSpin Tissue kit (Macherey-Nagel, Düren, Germany) 13 A to 13 J samples were centrifuged at 21,000 rcf for 20 min to pellet cells. The pellet was resuspended in 180 µl of Buffer 1 (B1) and 50 µl of 10 mg/ml lysozyme (Thermofisher, Waltham, USA), vortexed for 10 s and incubated at 37 °C for 1 h. Afterwards, 25 µl of Proteinase k and 20 µl of RNAse enzyme (20 mg/ml) (Thermofisher, Waltham, USA) were added to the samples followed by incubation at 56 °C for 2 h. Samples were then vortexed for 3 s and supplemented with 200 µl of the Buffer B3. This was followed by a vortex step for 1 min and incubation at 70 °C for 10 min in a thermoblock Techne DB-2 A Dri-Block Heater (Akribis Scientific Limited, Hereford, UK). Samples were vortexed for an additional 3 s followed by the addition of 210 µl of 96% -100% molecular grade ethanol and vortexing mixing for 1 min.

One NucleoSpin^®^ Tissue Column was placed into a Collection Tube for each sample. the sample was applied to the column and centrifuged for 1 min at 11,000 rcf. The Collection Tube with flowthrough was discarded and the column was placed into a new Collection Tube. This was followed by two silica membrane washes. Thereafter, 500 µl of Buffer BW was added and the samples were centrifuged for 1 min at 11,000 rcf. The flowthrough was discarded, and the column was placed back into the Collection Tube. Afterwards, 600 µl of Buffer B5 was added to the column, and centrifuged for 1 min at 11,000 rcf, the flowthrough was discarded, and the column was placed back into the Collection Tube. To dry the silica membrane, the column was centrifuged for 1 min at 11,000 rcf. Residual ethanol was removed during this step. To elute the pure DNA, the NucleoSpin^®^ Tissue Column was placed into a 1.5 ml tube and 100 µl Buffer BE was added, followed by incubation at room temperature for 1 min and centrifugation for 1 min at 11,000 rcf.

For the MN NucleoMag^®^ Pathogen kit (Macherey-Nagel, Düren, Germany). To lyse the sample, 20 µl Proteinase K, 180 µl of the Lysis Buffer NPL1, and 4 µl of the Carrier RNA stock solution was added to 200 µl of each sample. Samples were pipette mixed and incubated at 56 °C for 15 min with intermittent shaking. Following cell lysis, the samples were spun down to collect any residual sample from the lysis tube lids.

NucleoMag^®^ Beads were resuspended by vortexing and 20 µl resuspended beads was added to each sample lysate along with 600 µl Binding Buffer NPB2. Samples were briefly mixed by pipetting before further mixing on a Hula Mixer™ Sample Mixer (Thermo Fisher Scientific, Waltham, USA) for 5 min at room temperature. The magnetic beads were separated by placing the bead mixture on a magnetic DynaMag™-2 Magnet (Invitrogen™ Thermo Fisher Scientific, Waltham, USA). After 2 min, the supernatant was removed by pipetting without disturbing the beads on the magnetic side.

After removing the tubes from the magnetic stand, 600 µl of Buffer NPW3 was added and the beads were resuspended via shaking for 3 min to ensure complete resuspension. The tubes were placed back on the magnetic stand, incubated for 2 min and the supernatant was removed by pipetting. Following the first wash, the tubes were removed from the magnetic stand, 600 µl of the Buffer NPW4 was added and the beads were resuspended by shaking. Tubes were placed back on the magnetic stand and again, incubated for 2 min followed by the removal of the supernatant by pipetting. After removing the tubes from the magnetic stand, 600 µl of freshly prepared 80% molecular grade ethanol was added, and the beads were resuspended by shaking.

Samples were centrifuged for 5 s to collect all the fluid from the tube walls and then placed back in the magnetic stand, incubated for 2 min, followed by removal of the supernatant. A 10 µl extended tip was used to collect any remaining ethanol from the bottom of the tube and allowed to dry for 10 min at room temperature. Air-dried tubes were removed from the magnetic stand, supplemented with 50 µl of NPE5 (pre-heated to 56 °C) and resuspended by shaking for 5 min at 56 °C. Tubes were then placed back on the magnetic separator, and incubated for 2 min until all beads were attracted to the magnets. The resulting supernatant containing the purified nucleic acids was then transferred to a new 1.5 ml low-biding tube.

### Genomic DNA quality control

The DNA quality of all 140 samples was evaluated using the Nanodrop 2000c spectrophotometer (Thermo Fisher Scientific, Waltham, USA. For each measurement, 2 µl of DNA was analysed according to the manufacturer’s instructions. DNA purity was assessed using the A260/A280 and 260/230 absorbance ratio. The A260/A280 ratio is commonly used to evaluate protein contamination, with values around 1.8-2.0 generally indicating relatively pure DNA. The A260/A230 ratio reflects contamination by organic compounds such as polysaccharides, phenolic compounds, and other cellular metabolites that absorb at approximately 230 nm. Values close to ~ 2.0 are typically considered indicative of relatively pure nucleic acid preparations, while lower values may indicate co-purified organic contaminants.

For DNA quantification, the Qubit^®^ double-stranded DNA High Sensitivity (ds/HS) assay kit (Thermo Fisher Scientific, Waltham, USA) was used to estimate the DNA quantity in all samples, for which 2 µl DNA sample was used according to the manufactory instructions. The amount of DNA present estimated by the Qubit assay was multiplied by the volume of the sample to estimate the total amount of the DNA.

### Verification of long fragment DNA amplifiability

To confirm that the extracted DNA was suitable for downstream amplification-based applications, a PCR assay targeting the pncA genes was performed using primers pncA700 forward (5’-GCTGGTCATGTTCGCGATCG-3’) and pncA700 reverse (5’-CGCCGCCAACAGTTCATCC-3’), generating a 615 bp [24]. Amplification products were visualized with SYBR Safe-DNA Gel Stain (Thermofisher, Waltham, USA) after electrophoretic fractionation in 1% Agarose Gel in 1x Tris-Acetate EDTA (TAE) buffer (Thermofisher, Waltham, USA), at 95 V for 45 min. This assay was used solely to confirm PCR amplifiability of the extracted DNA and was not intended to assess high–molecular-weight DNA integrity.

### Whole-genome sequencing

From each extraction method, three samples were selected for library preparation based on their proximity to the mean total dsDNA concentration for that method. This approach aimed to select representative samples while minimizing the influence of extreme outliers that could bias sequencing performance comparisons. To enhance the library preparation quality, DNA purification was performed through a bead-clean-up using AMPure XP Bead-Based Reagent (Beckman Coulter, Brea, USA). After resuspending the AMPure XP beads using vortex, a 1:1 sample/bead dilution was performed, based on the sample volume. After adding the beads to the DNA samples, the suspension was mixed by slowly pipetting up and down 10 X (times) to avoid bubbles, followed by incubation at room temperature for 5 min.

The samples in 1.5 ml tubes were placed into the magnetic stand for 5 min to separate the DNA from the liquid. Without removing the tubes from the magnetic stand), the liquid was carefully removed without resuspending or touching the beads. Thereafter, 100 µL of freshly prepared 80% molecular grade ethanol was added on the opposite side of the beads without removing the tubes from the magnetic stand and underwent incubation for 30 s before removing the ethanol. This wash was repeated once. To remove possible remaining ethanol, a P10 pipette was used to remove any residual at the bottom of the tube.

Still, within the magnetic stand, the samples were incubated at room temperature for approximately 5 min until the tube and beads were fully dried. Afterwards, the tubes were removed from the magnetic stand and placed in a stand. Between 8 and 22 µL of NFW, depending on the initial sample concentration, was immediately added for DNA resuspension. Following, the samples were incubated at room temperature for 5 min and placed again on the magnetic stand for an additional 5 min of incubation at room temperature. The supernatant containing eluted DNA was transferred to a new tube and quantified using a Qubit^®^ DNA ds/HS kit (Thermo Fisher Scientific, Waltham, USA) according to the manufacturer’s instructions.

The library preparation was performed using the Illumina DNA Prep kit (Illumina, San Die-go, USA) according to the manufacturer’s instructions except for the use of 10 PCR cycles instead of 8. The libraries were quality controlled using Qubit^®^ DNA ds/HS assay kit to quantify the concentration of DNA fragments, and TapeStation High Sensitivity D5000 kit for fragment size analysis.

The sequencing was performed using an Illumina MiniSeq instrument (Illumina, San Die-go, USA). There was a batch of four to five samples per run using a mid-output cartridge (300 cycles), totalling six runs.

### Statistics analyses and data visualization

For bioinformatics analysis, the MAGMA v1.1.1 pipeline (https://github.com/TORCH-Consortium/MAGMA/tree/master ) was used [25, 26]. The interpretation regarding the coverage was: ≥ 50X a genome was analysable, nothing should be missed, ≥ 20X to < 50X a genome was analysable with nearly all minor variants detected, ≥ 10X to < 20X a genome was analysable but some minor variants may not be detected, ≥ 5X to < 10X a genome was analysable but some variants may not be detected and > 5X a sample was not analysable.

To ensure a fair comparison of results between different DNA extraction methods, a standardized median coverage of 5 million reads was established for the criteria used in this study 8. This standardization was determined based on the mapped percentage and insert size, using the formula [median coverage] divided by [raw reads] multiplied by 5,000,000.

Statistical analyses were performed using R software (v4.3.1). Descriptive statistics, including mean, median, standard deviation (SD), coefficient of variation (CV), and mean absolute deviation (MAD), were calculated for each DNA extraction method to summarize variability across technical replicates for DNA quantity and purity metrics.

Given the heterogeneous nature of the evaluated parameters, including laboratory performance metrics (DNA yield, purity, library preparation quality, and sequencing output) and operational variables (turnaround time and cost), the study was designed as a comparative benchmarking analysis rather than a hypothesis-testing experiment.

Additionally, the large number of evaluated extraction workflows (*n* = 14) combined with the limited number of samples subjected to WGS (three representative samples per method) would require extensive multiple-testing correction, substantially reducing statistical power and limiting interpretability of formal significance testing. Therefore, descriptive statistics were used to characterize method performance, while a multi-criteria scoring framework was applied to integrate technical and operational parameters. This framework allows comparison of extraction workflows in a manner consistent with practical laboratory decision-making for implementing WGS in TB diagnostics.

To enable a structured comparison of the evaluated DNA extraction methods, a multi-criteria scoring framework was developed based on key parameters relevant for WGS performance and laboratory implementation. Ten criteria were evaluated, encompassing DNA yield, DNA purity, PCR amplifiability, sequencing library quality, sequencing performance, turnaround time, and reagent cost (Table 1). Each criterion was scored on a standardized scale from 1 (lowest performance) to 5 (highest performance). When DNA extraction yielded no detectable genomic DNA or when samples failed library preparation quality control, the corresponding criteria were assigned the lowest score (score = 1). This approach ensured that extraction failures were incorporated into the comparative evaluation of methods.

**Table 1:** Criteria and scores to evaluate the DNA extraction methods presented in this study

Criteria	Score 1	Score 2	Score 3	Score 4	Score 5
1. Total dsDNA Quantity (ng) ^a^	< 5 ng or Undetected	5–9.9 ng	10–49 ng	50–100 ng	> 100 ng
2. DNA purity (Nanodrop 260/280) ^a^	< 1.0 or ≥ 3.0	> 2.5; < 3.0	1.0–1.49; > 2.0 ≤ 2.5	1.5–1.79	1.8–2.0
3. DNA purity (Nanodrop 260/230) ^a^	< 0.5 or > 2.2	0.1–0.49	0.5–0.99	1.0–1.49; > 2.2 ≤ 2.5	1.5–2.2
4. DNA fragment 600 bp (*pncA* PCR) ^b^	All bands absent	Faint band	1 band present	2 bands present	All bands present
5. Library concentration (ng/µl) ^b^	Undetected or < 0.5 /ng/uL	< 1 ng/µl; >0.5 ng/µl	1-4.9 ng/µl	5–10ng/µl	> 10 ng/µl
6. Library fragment size (bp)^b^	> 1000 bp or < 300 bp, or intensity peak close to 0	> 300 bp; < 400 bp	400 bp-500pb and 651 bp-1000pb (typical peak, moderate shoulder/tailing)	400 bp-500pb and 651 bp-1000pb (typical peak, minor shoulder/tailing)	500 bp − 650pb (typical peak)
7. WGS mapped %^b^	< 40%	≥ 40%, < 60%	≥ 60%, < 70%	≥ 70%, < 80%	≥ 80%
8. WGS median coverage 5 M reads^b^	< 50X	≥ 50X, < 70X	≥ 70X, < 90X	≥ 90X, < 110X	≥ 110X
9. Turnaround time^a, c^	> 8 h	> 3 h, ≤ 8 h	> 2 h, ≤ 3 h	> 1 h 30 min, ≤ 2 h	< 1 h 30 min
10. Cost (EUR)^d^	≥ 5€	> 3€ – 4€	> 2€ – 3€	1€ – 2€	< 1€

^a^Based on the mean of the 10 replicates for each method

^b^Based on the mean of the three replicates for each method which were selected based on the distribution of total double-strand (ds) DNA (ng)

^c^The estimated turnaround time was calculated using a daily work schedule of 8 h

^d^Cost per sample calculated initially in South African Rands (ZAR) and converted to euros (EUR)

Thresholds for each score were defined based on commonly accepted QC metrics for DNA extraction and Illumina library preparation, manufacturer guidelines, and empirical experience from TB genomics laboratories performing WGS on CPC. For DNA extraction metrics (e.g., dsDNA quantity and NanoDrop purity ratios), scores were calculated using the mean values from ten technical replicates per method. For sequencing-related parameters (library concentration, fragment size, mapping percentage, and genome coverage), scores were derived from the mean values of three representative samples selected based on their proximity to the mean DNA concentration distribution.

The individual scores for the ten criteria were summed to generate a composite performance score for each extraction method, with a maximum possible score of 50. All criteria were weighted equally to reflect the study’s implementation-oriented objective, where laboratory feasibility factors such as cost and turnaround time are considered as important as sequencing performance metrics. Radar plots were used to visualize the multi-dimensional performance profiles of each extraction method. The radar plot summarizes the performance of each extraction method across the ten evaluated criteria, allowing simuntaneous visualization of laboratory efficiency and sequencing performance (Supplementary Fig. 1).

## Results

### DNA yield and quality

**Fig. 2:**
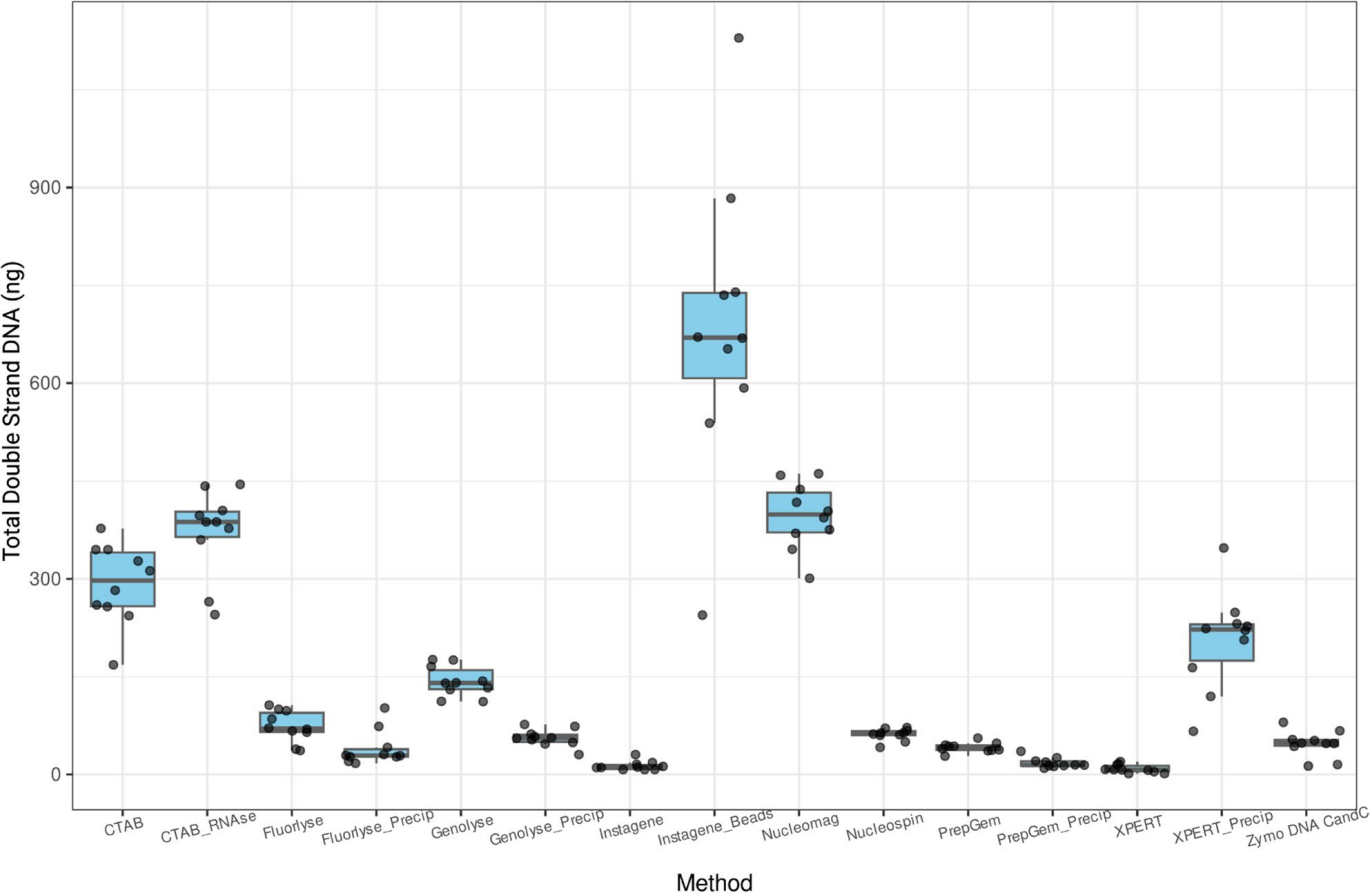
Total double-strand (ds) DNA (ng) distributed according to each DNA extraction method

The DNA quantity and purity results for each sample and DNA extraction method can be found in Supplementary Tables 1 and Supplementary Table 2. Among the DNA extraction methods used in this study, those that yielded higher amounts of DNA included IGM/RH, NucleoMag Pathogen, CTAB/RNAse, CTAB, Xpert/Precipitation, and GenoLyse/Precipitation being scored as 5 (Fig. 2). Although there was significant variability, the DNA yield achieved using these methods remained high. Of these, Xpert buffer/Precipitation exhibited some outlier samples with lower dsDNA concentrations, while NucleoMag Pathogen consistently provided higher and more stable DNA concentrations.

When examining the distribution of total dsDNA (in ng) for each method, a deviation from a normal distribution was observed, as seen in Supplementary Fig. 2. However, due to the lack of detectable genomic DNA from the samples 4 A to 4 J, which underwent the Zymo Quick-DNA Fungal/Bacterial Miniprep kit, it was not feasible to assess the quantity distribution in these cases.

While evaluating DNA extraction methods for nucleic acid purity using NanoDrop spectrophotometry, for the A260/A280 ratio measurements, the CTAB, CTAB/RNase, and FluoroLyse/Precipitation approaches, were closer to the desired threshold of 1.8 to 2.0 (Supplementary Fig. 3) and thus scored as 5. Conversely, the Zymo DNA Clean/Concentrator kit and GenoLyse showed ratios that deviated further from this range, especially due to the presence of outliers. Regarding the A260/A230 ratio, FluoroLyse/Precipitation and PrepGem/Precipitation presented values below the ideal threshold of 2.0, being scored as 5, however, due to the unequal distribution results and extended outliers, the judgment was based on the experience of working with the CPC.

When assessing the fragment size of the DNA obtained through *pncA* PCR and electrophoresis, it was observed that only the Xpert buffer/Precipitation and Xpert buffer/Purification DNA extraction methods did not show any bands, and they were scored as 1. In contrast, all other methods consistently displayed visible bands for the triplicate samples, earning them a score of 5.

### Downstream sequencing analysis

Triplicate samples located at the midpoint of the distribution, as previously emphasized, were selected for WGS library preparation and sequencing (Supplementary Fig. 4). Input DNA concentration ranged from 0.3 to 20 ng per sample (Supplementary Table 2). Among the 14 methods tested, two of them failed during genomic library QC, and as a result, were excluded from WGS. These methods are identified as follows: Xpert/CTAB purification (9 C, 9 F, 9 J) and Xpert/Ethanol Precipitation (11B, 11 C, 11E). In the case of Zymo kits, one sample of each kit failed library preparation QC: Zymo Clean and Concentrate kit (3G) and Zymo Quick-DNA Fungal/Bacterial Miniprep kit (4 A).

Concerning the criteria based on genomic library concentration (ng/µl), the lowest concentration was 2.32 ng/µl (PrepGem kit) and the highest was 16.25 ng/µl (NucleoSpin^®^ Tissue kit) while regarding the fragment size (bp) the lowest fragment was 465 bp (NucleoSpin^®^ Tissue kit) and the highest was 629 bp (Zymo Clean and Concentrate kit. When comparing the WGS output data, CTAB/RNase, Genolyse/Precipitation and Zymo Clean and Concentrate kit demonstrated were scored 5 based on criteria of mapped percentage with results of 80.17%, 81.21% and 80.10%, respectively. Regarding the coverage, Genolyse/Precipitation and Zymo Clean/Concentrate kits presented 110X/114x and scored 5. All scores are described in Supplementary Table 3.

### Turnaround time and cost

The turnaround time for processing sequenced samples varied from 1 h 20 min (IGM) to over 8 h (CTAB, CTAB/RNase) from the beginning of DNA extraction to its completion. Considering the scores, both CTAB and both Zymo kits methods scored 1 and both IGM kits and Xpert buffer/Precipitation were scored 5.

As for cost analysis, the values were initially calculated in ZAR for the year 2023, considering only reagent expenses, and subsequently converted to EUR, with an exchange rate of 1 EUR (€) = 20.488 ZAR (as of June 23, 2023). The cost per sample ranged from € 0.90 (IGM) to € 6.46 (PrepGEM Bacteria kit/Precipitation), being scored 5 and 1, respectively. Zymo Quick-DNA Fungal/Bacterial Miniprep kit also scored 1. Detailed turnaround time and cost data for each kit can be found in Supplementary Table 4.

### Radar chart combined criteria

**Fig. 3:**
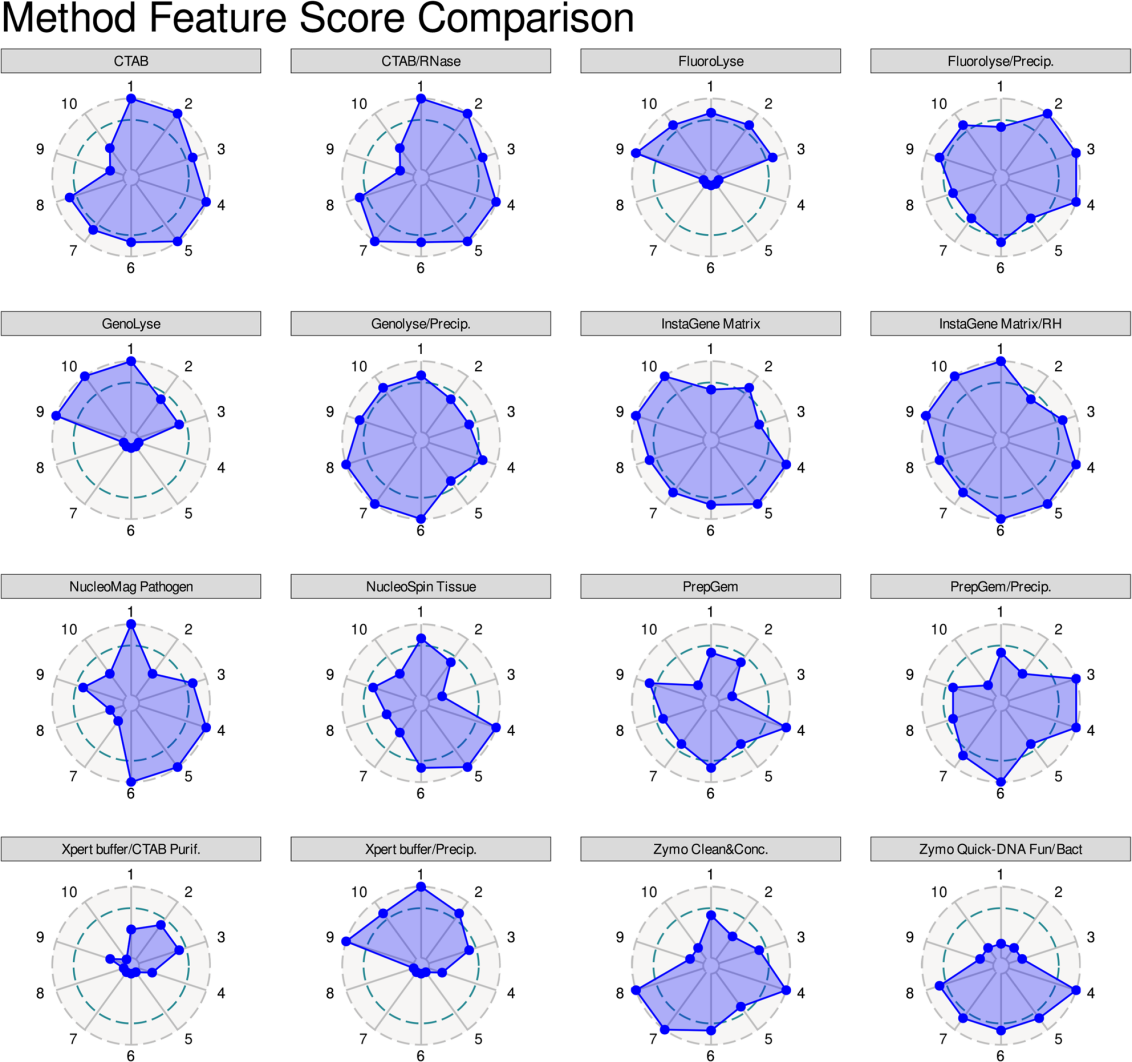
DNA Extraction methods scored according to the 10 criteria

The final scores for 10 criteria are described in Supplementary Tables 4 and graphically represented in Fig. 3. Considering the summarized scores across the 10 criteria for each method, the top five best-performing methods were as follows: InstaGene Matrix/RH (45), InstaGene Matrix (42), CTAB/RNase (40), GenoLyse/Precipitation (40), FluoroLyse/Precipitation (39), and CTAB (39). For the NucleoSpin Tissue kit and both Zymo kits, calculations for criteria 5, 6, 7, and 8 were carried out using duplicate data points. This was done in instances where the third sample was either undetected, leading to its removal from sequencing, or when the library concentration was around 0.2 ng/µl, also resulting in exclusion from sequencing to mitigate handling errors.

## Discussion

The successful application of WGS to guide clinical management and genomic surveillance of TB depends on obtaining sufficient, high-quality *Mtb* DNA from clinical samples in a manner that is reproducible, cost-effective, and compatible with diagnostic laboratory workflows. This study systematically compared 14 extraction methods on CPC, addressing not only DNA yield and purity but also library preparation success, sequencing quality, turnaround time, and cost. By benchmarking against the conventional CTAB method, we demonstrate that several commercial kits can achieve equivalent or superior sequencing results while offering practical advantages for routine use (Supplementary Tables 3 and 4; Fig. 3).

CPC samples contain low *Mtb* biomass and residual host or commensal DNA, which can complicate DNA extraction. A saline wash combined with DNase I digestion was therefore used to improve mycobacterial DNA purity, as previously described [18]. Among the evaluated methods, IGM/HSH [6, 12] achieved the highest overall performance score (45/50), followed by IGM alone and CTAB/RNase (Supplementary Table 3; Fig. 3). These methods consistently produced libraries with adequate fragment size, mapping percentages above 80%, and median genome coverage exceeding recommended thresholds for WGS analysis (Supplementary Table 2). The strong performance of IGM-based methods suggests that simple resin-based extraction combined with mechanical disruption can effectively lyse the lipid-rich mycobacterial cell wall while maintaining DNA integrity. Mechanical lysis likely contributes to improved DNA yield by increasing cell disruption efficiency, as previously reported for bead-beating–based extraction workflows [27–29]. In agreement with these studies [26–28, 27–29], mechanical disruption increased DNA yield in our dataset, with mean dsDNA concentrations increasing substantially when mechanical homogenization was applied (Fig. 2). Although CTAB remained one of the highest-performing methods in terms of DNA yield and sequencing metrics, its workflow required the longest processing time (> 8 h), limiting its practicality for high-throughput diagnostic laboratories [30].

Although spectrophotometric purity ratios are commonly used to assess DNA quality, our results indicate that they were poor predictors of sequencing success for CPC-derived DNA. Many samples showed A260/A230 values below the recommended ~ 2.0 threshold, consistent with co-purified organic compounds such as polysaccharides from the mycobacterial cell envelope. Despite this, these samples frequently produced high-quality sequencing libraries with adequate fragment size, mapping percentage, and genome coverage after AMPure bead clean-up. This suggests that moderate levels of co-purified contaminants do not necessarily compromise WGS when appropriate downstream purification steps are applied [29]. Because WGS was performed on only three samples per extraction method, the dataset lacks sufficient statistical power to formally evaluate associations between DNA purity metrics and sequencing outcomes. These observations suggest that spectrophotometric purity ratios should be interpreted cautiously for CPC-derived DNA and that sequencing performance provides a more relevant indicator of DNA suitability for WGS [31, 32].

DNA fragment size also plays an important role in library preparation and sequencing performance. In this study, most extraction methods produced fragments within the recommended range for Illumina library preparation (300–1000 bp), enabling successful sequencing on the MiniSeq platform. Interestingly, even samples with lower DNA input concentrations (e.g.: total DNA 10.95 ng from the IGM kit) successfully generated sequencing libraries, demonstrating the robustness of the Illumina DNA Prep workflow for CPC-derived DNA [33]. This finding is consistent with previous studies demonstrating that Illumina-based WGS can tolerate relatively low input DNA concentrations when library preparation and size selection steps are optimized.

Illumina library preparation guidelines recommend library concentrations of approximately 10–20 nM and fragment sizes between 300 and 1000 bp for optimal sequencing performance [34]. In this study, even the lowest library concentration (2.11 ng/µL, Zymo Clean and Concentrate kit) produced libraries with a fragment size of 629 bp, achieving 80.10% mapping and 114 X coverage. Some samples (CTAB, CTAB/RNase, and IGM) showed deviations from the ideal peak shape, including a shoulder on the right side of the fragment distribution. Despite these variations, these libraries still generated high-quality sequencing results (scores 4–5), highlighting the importance of accurate molarity calculations and quality assessment using fluorometric quantification and fragment analysis prior to sequencing. High genome coverage (> 110×) obtained with the GenoLyse/Precipitation and Zymo Clean and Concentrate methods demonstrates that robust genomic data can be generated from CPC samples using appropriate extraction workflows. In contrast, the NucleoMag^®^ Pathogen kit produced high DNA concentrations and acceptable purity ratios but resulted in low genome coverage (12X) and poor Mtb mapping (9.15%), indicating that DNA quantity alone does not reliably predict sequencing success. This observation highlights the need for WGS-specific validation of extraction methods. One possible explanation for the reduced performance of the NucleoMag^®^ Pathogen kit is the presence of carrier RNA in the extraction chemistry. Although carrier RNA can enhance nucleic acid recovery from low-biomass samples, it may interfere with downstream library preparation or compete with target DNA during sequencing. However, this hypothesis remains speculative and requires further investigation.

From an implementation perspective, several simplified extraction methods offer practical advantages for diagnostic laboratories. In particular, IGM was the most cost-effective method (<€1 per sample) while still producing acceptable sequencing results. Methods based on GenoLyse or FluoroLyse buffers also showed promising performance and may be particularly attractive for laboratories already performing line probe assays, as they can be integrated into existing workflows with minimal additional infrastructure requirements. These results highlight that extraction method selection should consider not only sequencing performance but also operational factors such as turnaround time, cost, and compatibility with existing laboratory workflows.

This study limitations are: (1) WGS was performed on only three representative samples per extraction method, limiting the ability to formally assess variability and failure rates across methods; (2) experiments were conducted in a single laboratory environment, and reproducibility across laboratories remains to be evaluated; (3) pooled CPC samples were used to standardize input material across extraction methods, which allowed controlled benchmarking but does not capture variability present in individual clinical specimens; (4) despite efforts to maintain sample homogeneity through thorough homogenization prior to aliquoting, variation in the number of *Mtb* cells between samples may still have occurred. Consequently, pipetting bias during sample distribution may have contributed to the variability observed in DNA yield and quality across some extraction methods, reflecting variability that may also occur in routine laboratory workflows; and (5) DNA integrity was not assessed using a fragment analyzer (e.g., TapeStation). Instead, library preparation success and WGS performance metrics were used as indicators of DNA suitability for sequencing. The PCR assay targeting a 615 bp *pncA* fragment was included only to confirm DNA amplifiability and does not provide a comprehensive assessment of long-fragment DNA integrity. More robust evaluation would require long-fragment PCR assays or long-read sequencing approaches such as Oxford Nanopore or PacBio.

Further evaluation using individual patient CPC samples with varying bacterial loads and culture conditions will therefore be important to assess real-world performance. Additional investigation of pre-extraction treatments and factors influencing method performance, including potential carrier RNA interference, may further refine extraction workflows. Despite these limitations, this study demonstrates that several simplified extraction methods can generate DNA suitable for WGS from CPC samples while substantially reducing cost and turnaround time compared with CTAB. These findings support the integration of practical, implementation-oriented extraction workflows into routine TB genomic surveillance programs.

## Conclusions

To assess DNA performance across various criteria, including DNA yield, purity, fragment size, library and whole-genome sequencing quality control, turnaround time, and cost, a comparative analysis of multiple DNA extraction methods was conducted. The findings revealed that the most effective commercial DNA extraction kits were IGM/HSH, IGM, GenoLyse/Precipitation, and FluoroLyse/Precipitation, outperforming the CTAB reference method. Notably, the inclusion of mechanical lysis methods such as HSH contributed to increased DNA yield.

Beyond technical performance, this study underscores the importance of DNA extraction methods that are compatible with scalable and potentially automated workflows, which are essential for integrating WGS into routine clinical and surveillance applications. While DNA yield and purity ratios provide initial quality indicators, the ability of extracted DNA to generate robust libraries and high-coverage genomes ultimately determines suitability for WGS. Notably, the inclusion of mechanical lysis methods such as HSH contributed to increased DNA yield.

## Supplementary Information

Supplementary Material 1.

## Data Availability

Sequence data that support the findings of this study have been deposited in the European Nucleotide Archive (ENA) with the accession code PRJEB102401.
