# Supplementary material for: Evaluation of DNA extraction methods from clinical Mycobacterium tuberculosis primary liquid culture for whole-genome sequencing
Source: BMC Genomics. 2026 May 19;27:605. doi: 10.1186/s12864-026-12812-w (PMC13352775; doi:10.1186/s12864-026-12812-w)
Supplement: Supplementary file 1 — Supplementary Material 1. [file 12864_2026_12812_MOESM1_ESM.zip › cpc_kits_suppl_material/cpc_kits_suppl_figures/cpc_kits_suppl_figs_v4/Supplementary Figure 2.pdf]

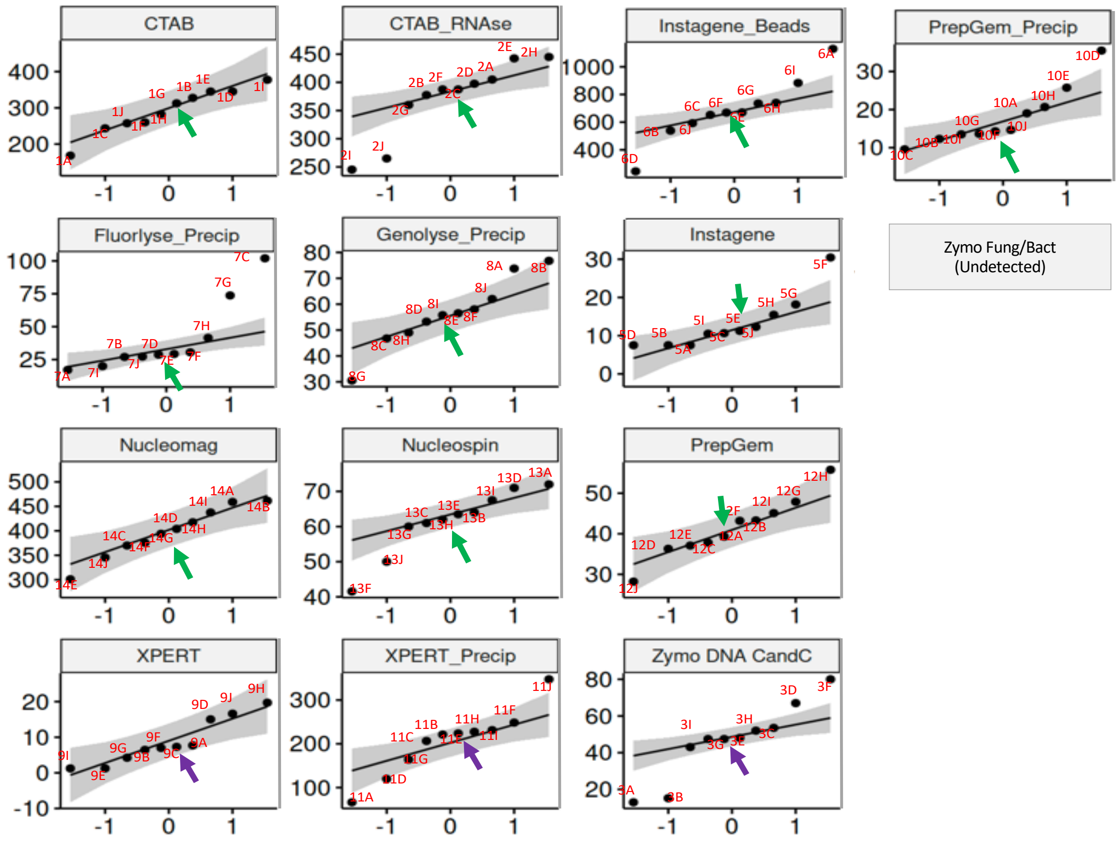

Supplementary Figure 2. Samples are categorised based on the total double-strand (ds) DNA quantity (measured in ng) for each DNA extraction method. Each dot on the plot corresponds to a specific sample and is labelled with its identification. Among these dots, the three samples highlighted in green that fell at the midpoint were chosen for the whole-genome sequencing (WGS) approach.
